# The innovative diagnostic model facilitates the differentiation between non - tuberculous mycobacterial lung disease and pulmonary tuberculosis

**DOI:** 10.3389/fcimb.2025.1667339

**Published:** 2025-10-31

**Authors:** Mingkun Qiao, Miao Li, Wenhui Qi, Lei Xu, Xiaohui Miao, Chao Cui

**Affiliations:** ^1^ Department of Thoracic Surgery, Haihe Hospital, Tianjin University, Tianjin, China; ^2^ TCM Key Research Laboratory for Infectious Disease Prevention for State Administration of Traditional Chinese Medicine, Tianjin, China; ^3^ Department of Obstetrics and Gynecology, Tianjin Medical University General Hospital, Tianjin, China

**Keywords:** non-tuberculous mycobacterial lung disease (NTM-LD), pulmonary tuberculosis lung disease (PTB-LD), differential diagnosis, nomogram, web-based scoring calculator

## Abstract

**Objectives:**

To construct a differential diagnostic model for Non-Tuberculous Mycobacterial Lung Disease (NTM-LD) and Pulmonary Tuberculosis Lung Disease (PTB-LD).

**Methods:**

Retrospective analysis of 300 NTM-LD and 300 PTB-LD patients (pathogen-confirmed) was performed. Patients were randomly split into training (2/3) and validation (1/3) sets. CT imaging, clinical data, and symptoms were analyzed. Logistic regression identified significant discriminative features, followed by random forest modeling to develop a diagnostic tool with web-based calculator. Model performance was validated using the independent validation set.

**Results:**

Univariate and multivariate analyses identified key discriminative factors (*P*<0.05): cough with sputum, hemoptysis, thin-walled cavities, centrilobular nodules, bronchiectasis, diabetes, and autoimmune diseases. The diagnostic model achieved 82.5% sensitivity and 85.5% specificity (ROC analysis), with validation showing 78% sensitivity and 85% specificity, confirming strong discriminative power and calibration.

**Conclusions:**

The model constructed based on patients’ CT imaging, basic clinical data, and symptomatic signs demonstrates commendable performance in the differential diagnosis of NTM-LD and PTB-LD, offering a convenient and practical auxiliary tool for clinical practice.

## Introduction

*Nontuberculous mycobacteria* (NTM) refer to a broad category of mycobacteria excluding the *Mycobacterium tuberculosis* complex and the *Mycobacterium leprae* complex. To date, approximately 200 species and 13 subspecies have been identified, most of which are opportunistic pathogens. These bacteria can invade the human body through the respiratory tract, gastrointestinal tract, skin, and other pathways, affecting multiple sites such as the lungs, skin and soft tissues, lymph nodes, and bones. Among these, the lungs are one of the most common sites of infection, leading to a condition known as nontuberculous mycobacterial lung disease (NTM-LD) (Kumar et al., 2024). In recent years, the incidence and prevalence of NTM-LD have been steadily increasing worldwide (Johansen et al., 2020). A 2024 epidemiological study confirmed that NTM constitute approximately 6.8% of sputum acid-fast bacilli smear-positive cases misdiagnosed as tuberculosis (Chen et al., 2024), underscoring significant risks of diagnostic errors and inappropriate therapeutic interventions. Moreover, the intrinsic resistance of NTM species to conventional anti-tuberculosis regimens necessitates protracted, multifaceted treatment protocols, which frequently entail severe drug-related toxicities and elevated treatment discontinuation rates. Consequently, NTM has emerged as a critical global threat to respiratory health. NTM-LD now represents not only a dominant etiology of chronic pulmonary infectious pathology but also a pivotal domain demanding intensified surveillance, advanced diagnostic methodologies, and targeted research initiatives.

NTM-LD often presents clinically with symptoms such as coughing with sputum hemoptysis, chest tightness, shortness of breath, low-grade fever, and night sweats. These symptoms are very similar to the clinical manifestations of pulmonary tuberculosis lung disease (PTB-LD) caused by infection with *Mycobacterium tuberculosis*. The clinical differentiation between NTM-LD and PTB-LD remains challenging due to similar manifestations. Current diagnostic workflows prioritize sputum smear microscopy, specifically Acid - Fast Bacilli (AFB) staining, as an initial screening tool for mycobacterial infections, but this method cannot distinguish NTM from *Mycobacterium tuberculosis* complex. Definitive diagnosis of NTM requires bacterial culture followed by species identification—a process that takes approximately 8 weeks under stringent biosafety protocols (Haworth et al., 2017). During this prolonged diagnostic period, patients with AFB-positive results are often empirically prescribed anti-tuberculosis therapy (ATT) to mitigate potential PTB transmission risks. However, this approach poses dual challenges: (1) overtreatment, exposing a subset of patients to unnecessary drug toxicity without clinical benefit, and (2) delayed targeted therapy, as most NTM species demonstrate intrinsic resistance to standard anti-tuberculosis agents, necessitating species-specific multidrug regimens distinct from PTB protocols (Conyers and Saunders, 2024). Therefore, establishing a stratified management system based on accurate differentiation between NTM-LD and PTB-LD holds significant clinical implications for achieving personalized diagnosis and treatment as well as optimal allocation of medical resources. With the advancement of analytical methods, the application of multifactorial mathematical models in the medical field has gradually expanded. Currently, numerous studies have explored the imaging-based differentiation between NTM-LD and PTB-LD, encompassing multiple modalities such as chest radiography and computed tomography (CT) (Xing et al., 2020; Park et al., 2023). However, existing research has predominantly focused on the discriminative value of individual imaging features, with limited systematic integration of clinical data (e.g., age, comorbidity profiles, and clinical symptoms) and quantitative imaging parameters for multidimensional analysis. Notably, there remains a paucity of exploration into integrating clinical-imaging multimodal indicators to construct mathematical prediction models. This study aims to evaluate the basic clinical data, symptoms and signs, quantitative CT imaging indicators, and comorbid conditions of NTM-LD and PTB-LD patients treated at our hospital since 2016. Through the development and validation of disease prediction models, we aim to provide predictive tools for early differential diagnosis of NTM-LD and PTB-LD, thereby enabling precision risk stratification with differentiated resource allocation and targeted interventions.

## Materials and methods

### Study subjects

A retrospective analysis was conducted on the basic clinical data, symptoms, signs, and imaging findings of patients with Non-tuberculous Mycobacterial Lung Disease (NTM-LD) and Pulmonary Tuberculosis Lung Disease (PTB-LD) admitted to the Department of Tuberculosis and Tuberculosis Surgery at Tianjin Haihe Hospital. The diagnosis of NTM-LD was based on the Chinese Guideline for the Diagnosis and Treatment of Non-tuberculous Mycobacterial Disease (2020 Edition) (C.M.A. Society of Tuberculosis, 2020), while PTB-LD was diagnosed in accordance with the Official Clinical Practice Guidelines jointly issued by the American Thoracic Society (ATS), Infectious Diseases Society of America (IDSA), and Centers for Disease Control and Prevention (CDC) (Hauk, 2018).microbiological diagnosis served as the “gold standard” for confirmation.

**Figure 1:**
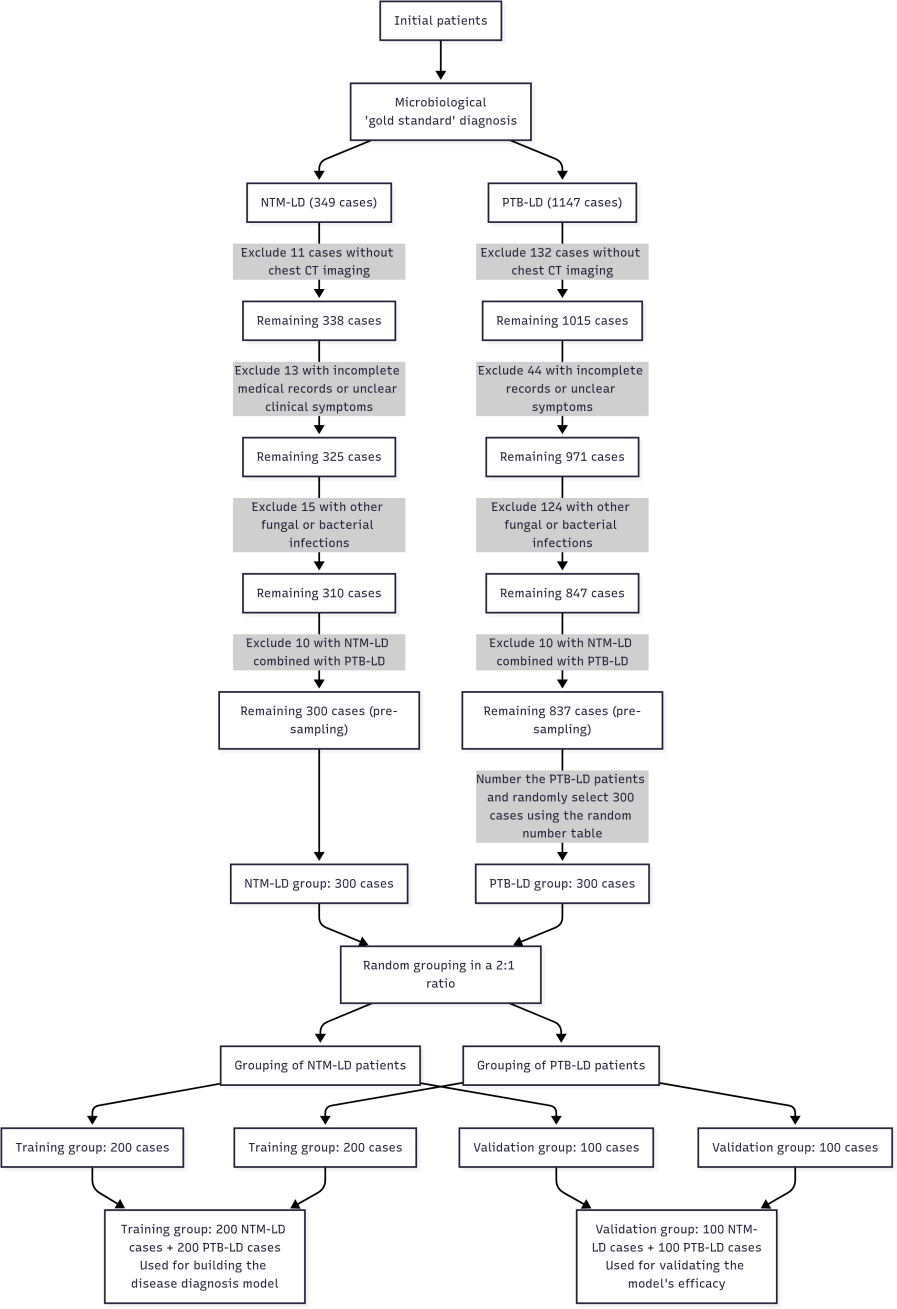
A flowchart depicting the patient grouping and data acquisition process Computed Tomography Scanning Method.

Given the low clinical incidence and relative rarity of NTM-LD, a 1:1 sample size ratio was employed to ensure balanced group sizes and enhance intergroup comparability—particularly to avoid insufficient statistical power due to an excessively small NTM-LD cohort. The NTM-LD group (case group) comprised 300 consecutive inpatients who met all inclusion and exclusion criteria and were admitted between June 2016 and June 2024. The PTB-LD group (control group) comprised 300 patients selected using the random number method from 837 hospitalized patients with PTB-LD who met both the diagnostic criteria and inclusion criteria during the same period. Subsequently, the 300 NTM-LD patients and 300 PTB-LD patients were separately and independently randomized: both cohorts were allocated to the training set and internal validation set at a 2:1 ratio using computer-generated random number tables (random sequences generated via SPSS 26.0 software). Specifically, the 300 NTM-LD patients were randomized into 200 cases in the training set and 100 cases in the internal validation set; similarly, the 300 PTB-LD patients were randomized into 200 cases in the training set and 100 cases in the internal validation set. The process of patient grouping and data collection is shown in **Figure 1**.

Inclusion criteria:

1. Definitive results of bacterial species identification and drug susceptibility testing.
2. Availability of authentic, complete, and standardized clinical diagnostic and treatment records.
3. Availability of complete chest CT imaging data.

Exclusion criteria:

1. Co-infection with other pulmonary diseases, such as bacterial or fungal infections.
2. Patients with concurrent NTM-LD and PTB-LD.

### Data quality control

To ensure the accuracy of research data, this study implemented rigorous quality control measures, detailed as follows:

Standardization and Reliability of Data Sources: Clinical data were uniformly sourced from electronic medical record systems, encompassing baseline information (age, gender), clinical symptoms (e.g., cough with sputum, fever), comorbidities (e.g., diabetes mellitus, autoimmune diseases), and microbiological test results. For imaging data, standardized equipment and parameters were strictly adopted to eliminate biases in imaging features arising from variations in device models or parameter settings. Blinded Imaging Assessment: Retrospective evaluations were conducted by three experienced radiologists, who remained blinded to patients’ microbiological outcomes. In cases of no consensus between two radiologists, the third radiologist’s assessment was incorporated to identify and document imaging features, facilitating quantitative analysis of indicators.

Data Management Rigor: A dedicated data management team from the Department of Tuberculosis, Tianjin Haihe Hospital, oversaw verification of data entry accuracy. This included standardizing terminology (e.g., “hemoptysis” “centrilobular nodules”) and systematically screening for outliers or contradictory data. All datasets were anonymized and encrypted for storage to safeguard patient privacy while ensuring traceability.

Unification and Standardization of Imaging Equipment: Our equipment was the Canon Aquilion Prime 64-slice spiral CT (Canon Medical Systems, Otawara, Japan). During the scan, patients were positioned supine with their hands elevated above their heads, entering the scanner headfirst, and standard protective measures were observed. The scanning parameters were set as follows: a tube voltage of 120 kV, automatic tube current modulation, a rotation time of 0.5 seconds per rotation, a matrix size of 512×512, and a collimator width of 64×0.5 mm. Image reconstruction was performed using both the FC30 (soft tissue algorithm) and FC52 (sharp algorithm), with a reconstruction slice thickness of 1mm and a slice interval of 0.8mm. The images were reviewed on the Canon workstation using lung window settings (1600 HU, -500 HU) and mediastinal window settings (400 HU, 40 HU).

### Data analysis

All data were analyzed using SPSS 26.0 statistical software. Categorical data were analyzed using the chi-square test, with the continuity-corrected chi-square test applied when expected frequencies were small. For variable selection in model construction, univariate and multivariate logistic regression analyses were employed with a stepwise strategy: first, variables with potential clinical significance (based on prior literature and expert consensus) and those showing marginal association in univariate analysis (*P*<0.10) were included as candidates; subsequently, multivariate logistic regression with backward elimination (likelihood ratio test) was performed to screen for independent predictors, where variables were retained if they met statistical significance (P<0.05) and contributed to model fitness as evaluated by Akaike information criterion (AIC). The “rms package” in R software (version: 4.4.0) was used to build the nomogram, and the Bootstrap resampling method (1000 samples) was applied to draw the calibration curve for internal validation. The receiver operating characteristic (ROC) curve was used to evaluate discriminative ability, the calibration curve to test calibration, and decision curve analysis (DCA) to assess clinical benefit. A *P*-value <0.05 was considered statistically significant.

## Result

### The demographic characteristics of the population

This study included a total of 300 NTM-LD patients and 300 PTB-LD patients. They were randomly divided into a training set (NTM-LD=200, PTB-LD=200) and an internal validation set (NTM-LD=100, PTB-LD=100) at a 2:1 ratio. There were no statistically significant differences in age and gender distribution between the two groups (*P* > 0.05). The demographic characteristics of the training set and internal validation set are shown in **Table 1**.

**Table 1:** Demographic characteristics of the training set and internal validation set.

Variables	Training set		*P-value*	Internal validation set		*P-value*
	NTM-LD	PTB-LD		NTM-LD	PTB-LD	
Age						
<45 years	35(17.5%)	50(25%)	0.067	19(19%)	29(29%)	0.098
≥45 years	165(82.5%)	150(75%)	81(81%)	71(71%)
Gender						
Male	131(65.5%)	139(69.5%)	0.393	55(55%)	65(65%)	0.149
Female	69(34.5%)	61(30.5%)	45(45%)	35(35%)

### Evaluation and selection of disease prediction model metrics in the training set

Variables for univariate analysis were selected based on expert consensus and clinical relevance. The study adopted a relatively lenient significance threshold (*P*<0.1) to allow inclusion of variables demonstrating “marginally significant” features in the multivariate analysis. Results revealed statistically significant differences between the two groups in the following 12 parameters (all *P*<0.1): age, cough with sputum production, hemoptysis, thin-walled cavities, centrilobular nodules, bronchiectasis, multi-lobar and multi-segmental involvement, exudative lesions, diabetes mellitus, autoimmune diseases, chronic obstructive pulmonary disease (COPD), and hematologic diseases. Conversely, no significant intergroup differences were observed in fever, wheezing, chest pain, disseminated lesions, fibrotic streaks, mediastinal lymph node enlargement, malignant tumors, interstitial lung disease, AIDS, pleural thickening, or hypoalbuminemia (all *P* > 0.1). Detailed data are presented in **Table 2**.

**Table 2:** Comparison of indicators between NTM-LD group and PTB-LD group.

Indicators	NTM-LD group (n=200)	PTB-LD group (n=200)	χ^2^/t	*P-value*
Symptoms				
Cough with sputum	177(88.5%)	110(55.0%)	55.367	<0.001*
Fever	88(44%)	75(37.5%)	1.750	0.186
Wheezing	79(39.5%)	69(34.5%)	1.073	0.300
Chest pain	24(12%)	15(7.5%)	2.301	0.129
Hemoptysis	81(40.5%)	20(10%)	49.286	<0.001*
Imaging manifestations				
Thin-walled cavities	132(66.0%)	78(39.0%)	95.837	<0.001*
Centrilobular nodules	135(67.5%)	38(19.0%)	66.828	<0.001*
Disseminated lesions	80(40%)	74(37%)	0.380	0.538
Bronchiectasis	116(58%)	26(13.0%)	88.438	<0.001*
Multi-lobar and multi-segmental involvement	155(77.5%)	139(69.5%)	3.286	0.070*
Exudative effusion	62(31%)	80(40%)	3.538	0.060*
Fibrous streaks	94 (47%)	84(42%)	1.012	0.314
Mediastinal lymph node enlargement	43(21.5%)	44(22%)	0.015	0.904
Comorbid conditions				
Diabetes mellitus	19(9.5%)	73(36.5%)	41.163	<0.001*
Autoimmune diseases	30(15.0%)	6(3.0%)	17.582	<0.001*
Chronic obstructive pulmonary disease	37(18.5%)	24(12%)	3.269	0.071*
Malignant tumors	13(6.5%)	9(4.5%)	0.770	0.380
Interstitial lung disease	37(18.5%)	29(14.5%)	1.161	0.281
AIDS	1(0.5%)	1(0.5%)	0	1
Hematologic diseases	51(25.5%)	67(33.5%)	3.077	0.079*
Pleural thickening	72(36.5%)	81(40.5%)	0.857	0.354
Hypoalbuminemia	65(32.5%)	67(33.5%)	0.045	0.832

“*” indicates *P*<0.1.

**Figure 2:**
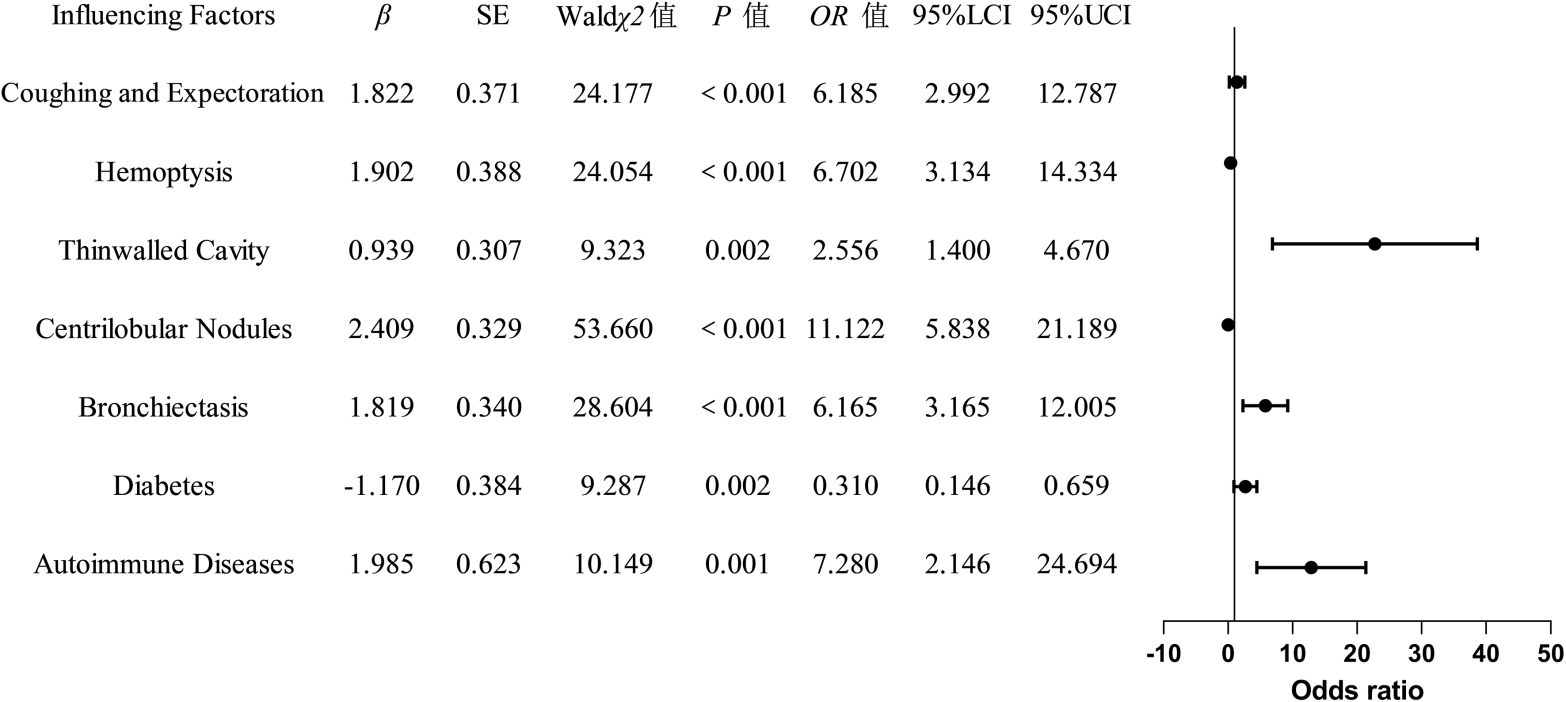
Forest Plot of Logistic Regression Analysis.

The indicators with statistically significant differences in the univariate analysis were included as independent variables in the multivariate logistic regression analysis. The final univariate analysis revealed that age, cough with sputum, hemoptysis, thin-walled cavities, centrilobular nodules, bronchiectasis, multi-lobar and multi-segmental involvement, exudation, diabetes mellitus, autoimmune diseases, chronic obstructive pulmonary disease, and hematological diseases were entered into the multivariate regression analysis. The multivariate analysis demonstrated that cough with sputum, hemoptysis, thin-walled cavities, centrilobular nodules, bronchiectasis, diabetes mellitus, and autoimmune diseases were independent risk factors for distinguishing between NTM-LD (nontuberculous mycobacterial lung disease) and PTB-LD (pulmonary tuberculosis lung disease), and these variables were ultimately incorporated into the model (**Table 3**, **Figure 2**). The nomogram model was constructed based on the following equation: Y = -3.650 + (1.822 × cough with sputum) + (1.902 × hemoptysis) + (0.939 × thin-walled cavities) + (2.409 × centrilobular nodules) + (1.819× bronchiectasis) + (-1.170 × diabetes mellitus) + (1.985 × autoimmune diseases). The probability *P* was calculated as: *P*=1/(1+exp(-Y)).

**Table 3:** Multivariate logistic regression analysis of factors influencing the classification between NTM-LD and PTB-LD.

Indicators	*β*	SE	Wald*χ2*	*P*	*OR*	95%CI	
						Low	High
Age	-0.797	0.434	3.372	0.066	0.451	0.193	1.055
Cough with sputum	1.822	0.371	24.177	<0.001**	6.185	2.992	12.787
Hemoptysis	1.902	0.388	24.054	<0.001**	6.702	3.134	14.334
Thin-walled cavities	0.939	0.307	9.323	0.002**	2.556	1.400	4.670
Centrilobular nodules	2.409	0.329	53.660	<0.001**	11.122	5.838	21.198
Bronchiectasis	1.819	0.340	28.604	<0.001**	6.165	3.165	12.005
Multi-lobar and multi-segmental involvement	0.080	0.371	0.047	0.829	1.084	0.524	2.242
Exudative effusion	0.110	0.332	0.109	0.741	1.116	0.582	2.141
Diabetes mellitus	-1.170	0.384	9.287	0.002**	0.310	0.146	0.659
Autoimmune diseases	1.985	0.623	10.149	0.001**	7.280	2.146	24.694
Chronic obstructive pulmonary disease	0.084	0.410	0.042	0.837	1.088	0.487	2.431
Hematologic diseases	-0.436	0.345	1.600	0.206	0.646	0.329	1.271

“**” indicates P < 0.05.

### Development and validation of a clinical prediction model for differentiating NTM-LD and PTB-LD

**Figure 3:**
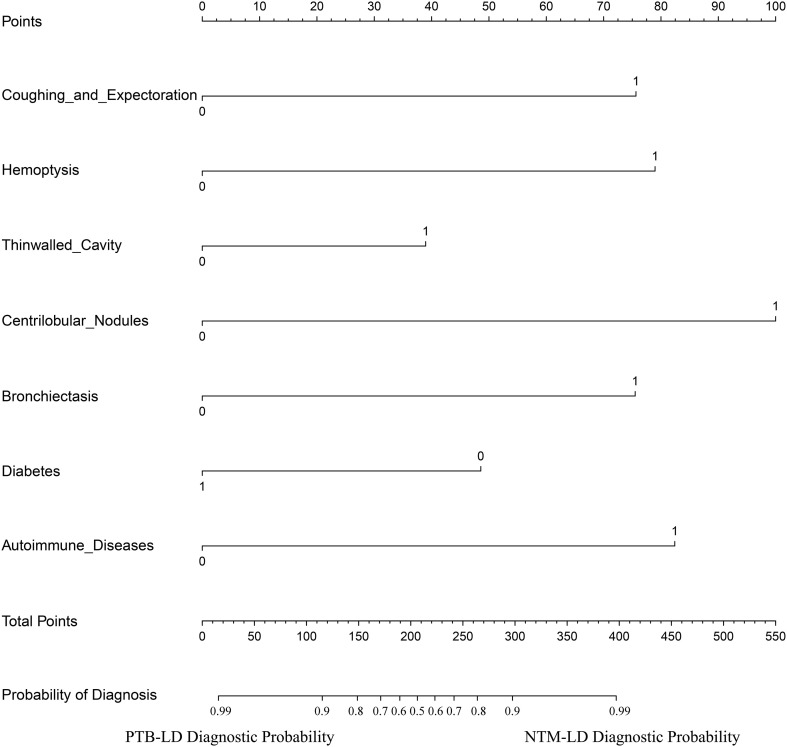
Nomogram prediction model for differentiating NTM-LD from PTB-LD.

**Figure 4:**
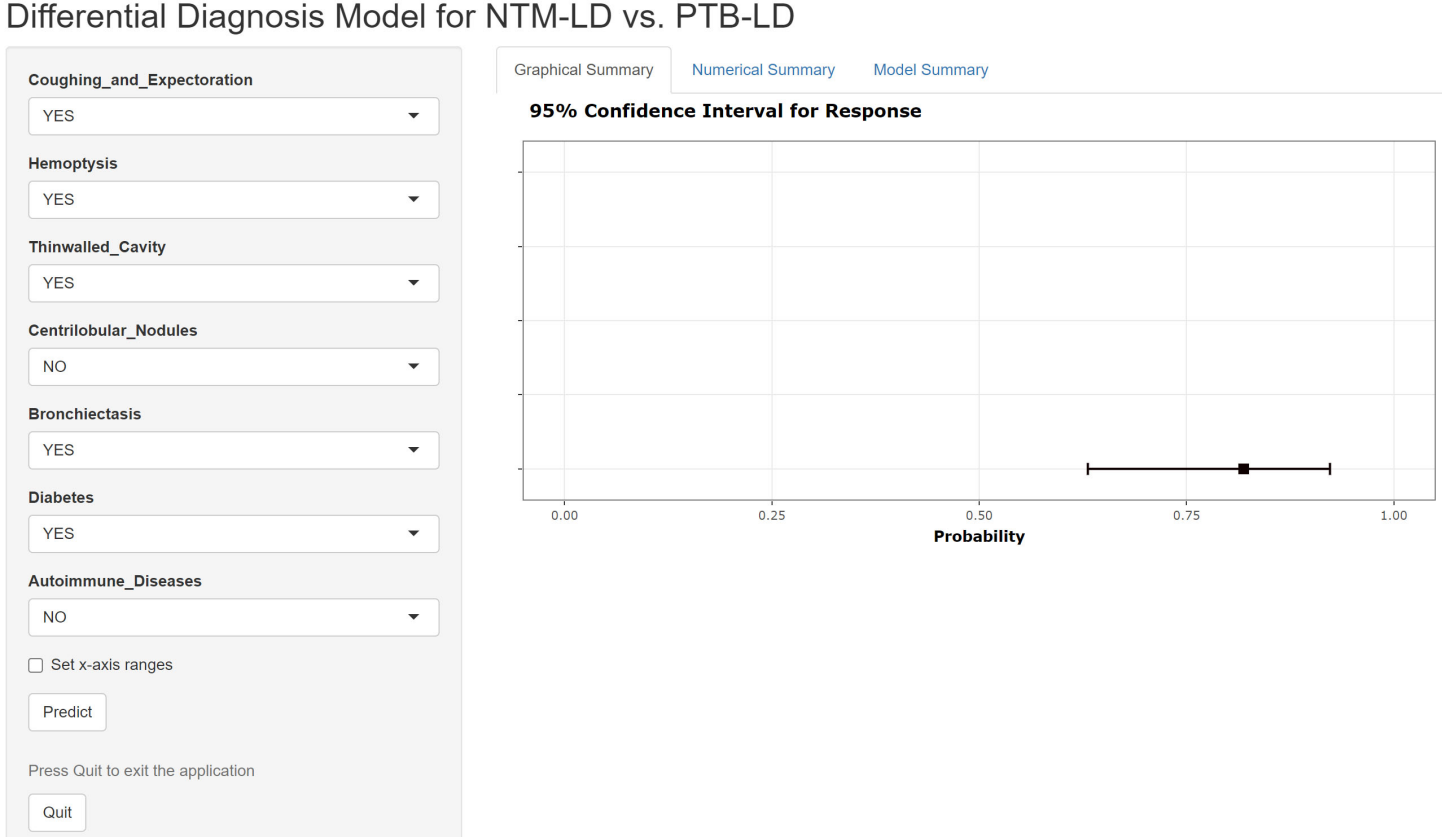
Web-based calculator for the differential model of NTM-LD and PTB-LD.

The constructed equation was visualized using the ‘rms package’ in R software to generate a nomogram prediction model for differentiating NTM-LD and PTB-LD (**Figure 3**). Based on the patient’s clinical manifestations, including cough with sputum, hemoptysis, thin-walled cavities, centrilobular nodules, bronchiectasis, Diabetes mellitus, and autoimmune diseases, values are assigned to each variable. By creating vertical lines on the nomogram, corresponding scores can be identified on the scoring axis for each parameter. These scores are summed to calculate the total score. Finally, the total score is projected onto the risk axis to estimate the probability of NTM-LD occurrence for that patient. For example, a patient presenting with cough and sputum receives a score of 75, while the absence of this symptom yields 0 points. Patients with hemoptysis are assigned 80 points, whereas those without receive 0. The presence of thin-walled cavities adds 40 points, and their absence contributes 0. Centrilobular nodules are scored 100 if present and 0 if absent. Bronchiectasis corresponds to 75 points when present and 0 when absent. Notably, patients without diabetes are assigned 50 points, while those with diabetes receive 0. Conversely, patients with autoimmune diseases are scored 80 points, and those without are assigned 0. These individual scores are summed to calculate the total score. The total score is then projected onto the risk axis of the nomogram to determine the patient’s probability of developing NTM-LD. To facilitate clinical use, we further developed a web-based score calculator based on this nomogram prediction model (accessible at https://dynamic-diagram.shinyapps.io/DynNomapp/), **Figure 4**.

**Figure 5:**
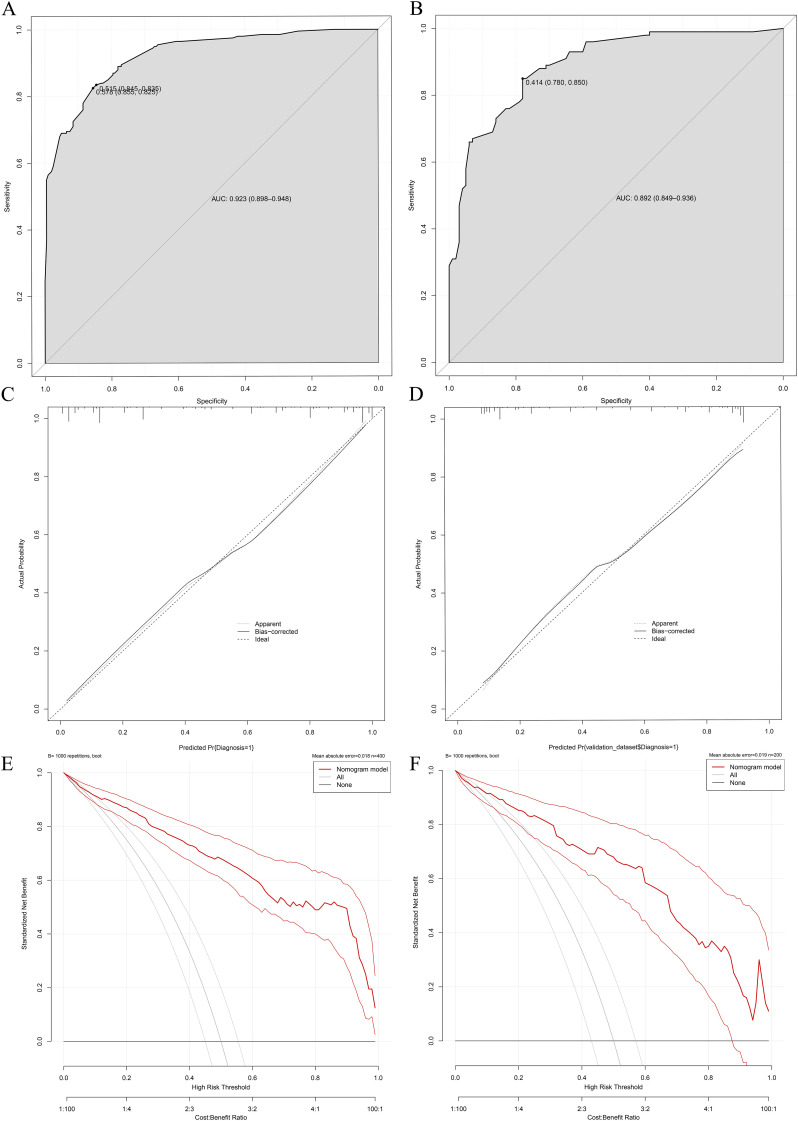
Performance validation of the differential diagnostic model for NTM-LD and PTB-LD. **(A, B)** Training/validation cohort ROC curves; **(C, D)** Training/validation cohort calibration curves; **(E, F)** Training/validation cohort decision curve analyses.

The validation results demonstrated consistent diagnostic performance of the nomogram across both the training and internal validation cohorts. In the training cohort, the model achieved an area under the receiver operating characteristic (ROC) curve (AUC) of 0.923 (95% confidence interval [CI]: 0.898-0.948), with a specificity of 85.5% and sensitivity of 82.5% (**Figure 5A**), indicating superior differentiation capability between NTM-LD and PTB-LD. Furthermore, calibration curve analysis revealed close agreement between predicted probabilities and observed outcomes confirming excellent calibration accuracy (**Figure 5C**). To further evaluate the model’s generalizability, an internal validation cohort comprising 100 NTM-LD patients and 100 PTB-LD patients was included. The model demonstrated correct diagnosis rates of 88.0% (88/100) for NTM-LD and 75.0% (75/100) for PTB-LD, achieving an overall diagnostic accuracy of 81.5% (163/200). In the validation cohort, the AUC reached 0.892 (95% CI: 0.792-0.900) with specificity of 78% and sensitivity of 85% (**Figure 5B**), indicating clinically generalizable discriminative performance. The Hosmer-Leme show test revealed no significant deviation between predicted and observed values (χ²=6.32, *P*=0.176), while the calibration curve exhibited 94.7% concordance with the ideal curve (**Figure 5D**), further validating calibration robustness. To quantify clinical utility, decision curve analysis (DCA) was employed to assess model net benefit. Across both training and validation sets, the model demonstrated significant clinical net benefit compared to “all- NTM-LD” or “all-PTB-LD” strategies when threshold probabilities across a wide range (**Figures 5E, F**), suggesting its applicability across diverse clinical decision-making scenarios.

## Discussion

Differentiating NTM-LD from PTB-LD remains a significant diagnostic challenge in respiratory medicine. This study developed a high-performance predictive model by integrating multidimensional indicators, including clinical characteristics, radiological patterns, and comorbidities. Furthermore, we designed an interactive web-based calculator to visualize the model’s outputs, providing a novel tool for early clinical discrimination between these two entities.

This study revealed distinct clinical profiles between NTM-LD and PTB-LD. Specifically, patients with NTM-LD demonstrated a significantly higher prevalence of chronic cough with sputum production (OR=6.185, *P*<0.001) and hemoptysis (OR=6.702, *P*<0.001), which aligns with the airway injury patterns in NTM-LD reported by Youssefnia A.et al (Youssefnia et al., 2022). The virulence of NTM is relatively lower compared to *Mycobacterium tuberculosis*, yet their enhanced adhesive capacity enables persistent colonization of airway mucosa. Characterized by unique lipid-rich cell wall components that resist phagocytic clearance and impede their elimination, NTM induce chronic airway inflammation through sustained antigenic stimulation. This pathological process promotes excessive mucus secretion and consequently exacerbates clinical manifestations of cough with sputum production (Schiff et al., 2019). Furthermore, NTM-triggered inflammatory responses disrupt vascular integrity in bronchial walls, resulting in enhanced vascular permeability. Concurrently, airway smooth muscle hyperreactivity and bronchoconstriction elevate intraluminal pressure, which synergistically exacerbates microvascular damage and significantly increases the risk of hemoptysis (Zhang et al., 2019). Moreover, our study identified several distinct imaging biomarkers-bronchiectasis, centrilobular nodules, and thin-walled cavities-that collectively reflect the unique pathophysiological features of nontuberculous mycobacterial lung disease (NTM-LD). The strong association between bronchiectasis and NTM-LD (OR=28.604, *P*<0.001) further supports the predominantly airway-centered, chronic inflammatory nature of this condition. Bronchiectasis is both a predisposing factor and a consequence of NTM infection (Retuerto-Guerrero et al., 2024). Structural airway damage facilitates persistent bacterial colonization, while chronic inflammation from NTM infection exacerbates mucociliary dysfunction and smooth muscle hyperreactivity, leading to progressive airway dilation—a self-perpetuating cycle known as the “vicious cycle hypothesis” in chronic respiratory diseases (Dartois and Dick, 2024). Notably, our findings align with those of Chu et al (Chu et al., 2015), highlighting its utility as a distinguishing imaging feature.We also observed a significantly higher prevalence of centrilobular nodules in NTM-LD (OR=11.122, *P*<0.001), consistent with findings by Iakobachvili et al (Iakobachvili et al., 2022). These nodules arise from bronchial spread of infection along the airway tree and represent early inflammatory foci within terminal bronchioles, typically appearing as “tree-in-bud” opacities on high-resolution computed tomography (HRCT). This pattern is widely recognized as a hallmark of NTM-LD, reflecting pathogen colonization of the airway epithelium and localized granulomatous inflammation without extensive tissue necrosis. Another key imaging feature in NTM-LD was the presence of thin-walled cavities (OR=2.556, *P*=0.002), which are believed to develop secondarily to long-standing bronchiectasis and parenchymal remodeling. These cavities are typically small, multiple, and predominantly located in the upper lobes, especially in the subclavicular and middle lobe regions. This observation is strongly supported by prior HRCT studies demonstrating that thin—walled or even “bubbly”—appearing cavities are more common in NTM-LD than in PTB-LD (Evans et al., 1996). Comparative analysis of underlying comorbidities revealed distinct pathophysiological predispositions: the NTM-LD cohort exhibited heightened susceptibility to autoimmune disorders (OR=7.280, *P*=0.001), whereas diabetes mellitus predominated in PTB-LD patients (OR=0.310, *P*=0.002), a dichotomy reflecting pathogen-specific modulation of immune microenvironmental susceptibility. The indolent and chronic progression of NTM infection drives sustained low-grade activation of the host immune system, disrupting the homeostatic immune regulatory network. This persistent immunostimulation compromises immune cell-mediated recognition and tolerance mechanisms toward self-antigens, thereby triggering aberrant autoimmune responses against host tissues and elevating susceptibility to autoimmune disorders (Liu et al., 2023). The elevated prevalence of diabetes mellitus in PTB-LD patients is mechanistically attributed to the hyperglycemic microenvironment serving as a nutritional reservoir that facilitates *Mycobacterium tuberculosis* proliferation, survival, and dissemination. Moreover, hyperglycemia affects cell metabolism, inhibits the functions of immune cells such as macrophages, reduces the body’s phagocytic and killing ability against *Mycobacterium tuberculosis*, and increases the risk of infection (Kamei et al., 2023).

The predictive model constructed based on the aforementioned key differences demonstrated favorable diagnostic performance, with a training cohort AUC of 0.923 (95% CI: 0.898-0.948), validation cohort AUC of 0.892 (95% CI: 0.849-0.936), and overall accuracy of 81.5%. It significantly outperforms existing models: Liu et al’s (Liu et al., 2023). chest X-ray DenseNet model (internal/external AUC: 0.86 ± 0.006/0.64 ± 0.017), Hu et al.’s (Hu et al., 2025) radiomics model (AUC 90.2%), and CT-only models—all limited by single-modality reliance, neglect of clinical context (e.g., symptoms like cough with sputum/hemoptysis and comorbidities), inability to capture clinical heterogeneity, and smaller sample sizes. By integrating clinical variables with imaging features, this model overcomes single-modality limitations, achieving superior discriminative power (higher AUC than comparative models), robust calibration (94.7% alignment of calibration curve with ideal reference; Hosmer-Lemeshow test, *P*=0.176), and confirming clinical net benefit via decision curve analysis-outperforming “treat-alls” or “no-intervention” strategies across threshold probabilities. It provides a reliable tool for precise differentiation between NTM-LD and PTB-LD, aiding in reducing misdiagnoses and optimizing patient management.

Furthermore, the transparent logistic regression-based algorithm employed in our model offers better interpretability for clinical practitioners compared to complex deep learning architectures, facilitating its potential integration into routine clinical decision-making processes. The innovatively developed web-based calculator (accessible at https://dynamic-diagram.shinyapps.io/DynNomapp/) facilitates clinical translation of our model by enabling physicians to obtain real-time personalized risk assessments through the input of eight binary variables. This tool supports clinical decision-making by stratifying patients according to their probability of NTM-LD. Individuals predicted as high-risk should avoid unnecessary anti-tuberculosis therapy (ATT) and instead undergo timely NTM-specific diagnostic testing—such as sputum culture for nontuberculous mycobacteria, species identification, NTM specific PCR, or metagenomic next-generation sequencing (mNGS)—to confirm the diagnosis and guide appropriate management. In contrast, patients classified as low-risk may be considered for early initiation of ATT if clinical and radiological features suggest pulmonary tuberculosis, provided that microbiological workup is concurrently pursued to confirm or exclude PTB-LD. The current version of the application represents a proof-of-concept implementation, designed to demonstrate the feasibility of translating our predictive model into a practical clinical decision-support tool. However, we acknowledge that formal usability testing and integration into routine clinical workflows have not yet been conducted. Future studies should evaluate the tool’s acceptability, efficiency, and impact on clinical decision-making through structured user experience assessments—such as System Usability Scale (SUS) surveys or cognitive task analyses—among frontline healthcare providers. Furthermore, pilot implementation in outpatient respiratory or infectious disease clinics could provide valuable insights into its compatibility with existing electronic health record (EHR) systems and standard diagnostic pathways.

This study has several limitations. First, the single-center, retrospective design may introduce selection bias and limit the generalizability of the findings to broader populations. Second, the relatively modest sample size (n=600) constrains statistical power for comprehensive subgroup analyses, particularly for rare clinical phenotypes. Furthermore, although multiple statistical and validation techniques were employed to minimize overfitting—such as internal bootstrapping and stepwise variable selection—some degree of model overfitting may still persist. To address these limitations, we plan to conduct a prospective, multi-center cohort study to externally validate the model’s performance across diverse healthcare settings and patient populations. Independent validation in larger, geographically representative cohorts will be critical to confirm its robustness, transportability, and clinical utility.

## Conclusion

The developed NTM-LD/PTB-LD differentiation model demonstrates significant clinical utility for optimizing diagnostic workflows. The implementation of its web-based tool (accessible at https://dynamic-diagram.shinyapps.io/DynNomapp/) could transform current diagnostic paradigms by reducing reliance on invasive procedures, while providing real-time decision-making support for precision antimicrobial therapy. Future efforts will focus on clinical validation and global dissemination of this tool to enhance tuberculosis management in resource-variable settings.

## Data Availability

The original contributions presented in the study are included in the article/**Supplementary Material**. Further inquiries can be directed to the corresponding author.

## References

[B1] Chen S. Zhong J. Yang Q. Song X. Zhang L. Ruan G.. (2024). Comparative analysis of non-tuberculous mycobacterial lung disease and lung colonization: a case-control study. BMC Infect. Dis. 24, 1159. doi: 10.1186/s12879-024-10067-y, PMID: 39407161 PMC11476636

[B2] Chu H. Q. Li B. Zhao L. Huang D. D. Zhang Z. M. Xu J. F.. (2015). Chest imaging comparison between non-tuberculous and tuberculosis mycobacteria in sputum acid fast bacilli smear-positive patients. Eur. Rev. Med. Pharmacol. Sci. 19, 2429–2439. doi: 10.1183/13993003.congress-2015.PA2674, PMID: 26214779

[B3] C.M.A. Society of Tuberculosis (2020). Guidelines for diagnosis and treatment of nontuberculous mycobacteriosis (2020 edition). Chin. J. Tuberculosis Respir. Dis. 43, 918–946. doi: 10.3760/cma.j.cn112147-20200508-00570

[B4] Conyers L. E. Saunders B. M. (2024). Treatment for non-tuberculous mycobacteria: challenges and prospects. Front. Microbiol. 15, 1394220. doi: 10.3389/fmicb.2024.1394220, PMID: 38887711 PMC11180805

[B5] Dartois V. Dick T. (2024). Therapeutic developments for tuberculosis and nontuberculous mycobacterial lung disease. Nat. Rev. Drug Discov. 23, 381–403. doi: 10.1038/s41573-024-00897-5, PMID: 38418662 PMC11078618

[B6] Evans A. J. Crisp A. J. Hubbard R. B. Colville A. Evans S. A. Johnston I. D. (1996). Pulmonary Mycobacterium kansasii infection: comparison of radiological appearances with pulmonary tuberculosis. Thorax 51, 1243–1247. doi: 10.1136/thx.51.12.1243, PMID: 8994523 PMC472771

[B7] Hauk L. (2018). Tuberculosis: guidelines for diagnosis from the ATS, IDSA, and CDC. Am. Family physician 97, 56–58., PMID: 29365230

[B8] Haworth C. S. Banks J. Capstick T. Fisher A. J. Gorsuch T. Laurenson I. F.. (2017). British Thoracic Society guidelines for the management of non-tuberculous mycobacterial pulmonary disease (NTM-PD). Thorax 72, ii1–ii64. doi: 10.1136/thoraxjnl-2017-210929, PMID: 29054853

[B9] Hu Y. Zhong L. Liu H. Ding W. Wang L. Xing Z.. (2025). Lung CT-based multi-lesion radiomic model to differentiate between nontuberculous mycobacteria and Mycobacterium tuberculosis. Med. Phys. 52, 1086–1095. doi: 10.1002/mp.17537, PMID: 39607908

[B10] Iakobachvili N. Leon-Icaza S. A. Knoops K. Sachs N. Mazères S. Simeone R.. (2022). Mycobacteria-host interactions in human bronchiolar airway organoids. Mol. Microbiol. 117, 682–692. doi: 10.1111/mmi.14824, PMID: 34605588 PMC9298242

[B11] Johansen M. D. Herrmann J. L. Kremer L. (2020). Non-tuberculous mycobacteria and the rise of Mycobacterium abscessus. Nature reviews. Microbiology 18, 392–407. doi: 10.1038/s41579-020-0331-1, PMID: 32086501

[B12] Kamei R. Sawahata M. Nakayama M. Yamada T. Taniguchi N. Bando M.. (2023). Prevalence of systemic and local risk factors for pulmonary non-tuberculous mycobacterial disease in Japan: a single-institution study. J. Rural medicine: JRM 18, 168–174. doi: 10.2185/jrm.2023-001, PMID: 37448701 PMC10336341

[B13] Kumar K. Ponnuswamy A. Capstick T. G. Chen C. McCabe D. Hurst R.. (2024). Non-tuberculous mycobacterial pulmonary disease (NTM-PD): Epidemiology, diagnosis and multidisciplinary management. Clin. Med. (London England) 24, 100017. doi: 10.1016/j.clinme.2024.100017, PMID: 38387207 PMC11024839

[B14] Liu Q. Du J. An H. Li X. Guo D. Li J.. (2023). Clinical characteristics of patients with non-tuberculous mycobacterial pulmonary disease: a seven-year follow-up study conducted in a certain tertiary hospital in Beijing. Front. Cell. infection Microbiol. 13, 1205225. doi: 10.3389/fcimb.2023.1205225, PMID: 37424783 PMC10325861

[B15] Liu C. J. Tsai C. C. Kuo L. C. Kuo P. C. Lee M. R. Wang J. Y.. (2023). A deep learning model using chest X-ray for identifying TB and NTM-LD patients: a cross-sectional study. Insights into Imaging 14, 67. doi: 10.1186/s13244-023-01395-9, PMID: 37060419 PMC10105818

[B16] Park M. Lee Y. Kim S. Kim Y. J. Kim S. Y. Kim Y.. (2023). Distinguishing nontuberculous mycobacterial lung disease and Mycobacterium tuberculosis lung disease on X-ray images using deep transfer learning. BMC Infect. Dis. 23, 32. doi: 10.1186/s12879-023-07996-5, PMID: 36658559 PMC9854086

[B17] Retuerto-Guerrero M. López-Medrano R. de Freitas-González E. Rivero-Lezcano O. M. (2024). Nontuberculous mycobacteria, mucociliary clearance, and bronchiectasis. Microorganisms 12 (4), 665. doi: 10.3390/microorganisms12040665, PMID: 38674609 PMC11052484

[B18] Schiff H. F. Jones S. Achaiah A. Pereira A. Stait G. Green B. (2019). Clinical relevance of non-tuberculous mycobacteria isolated from respiratory specimens: seven year experience in a UK hospital. Sci. Rep. 9, 1730. doi: 10.1038/s41598-018-37350-8, PMID: 30741969 PMC6370870

[B19] Xing Z. Ding W. Zhang S. Zhong L. Wang L. Wang J.. (2020). Machine learning-based differentiation of nontuberculous mycobacteria lung disease and pulmonary tuberculosis using CT images. BioMed. Res. Int. 2020, 6287545. doi: 10.1155/2020/6287545, PMID: 33062689 PMC7545409

[B20] Youssefnia A. Pierre A. Hoder J. M. MacDonald M. Shaffer M. J. B. Friedman J.. (2022). Ancillary treatment of patients with lung disease due to non-tuberculous mycobacteria: a narrative review. J. Thorac. Dis. 14, 3575–3597. doi: 10.21037/jtd-22-410, PMID: 36245600 PMC9562528

[B21] Zhang Z. X. Cherng B. P. Z. Sng L. H. Tan Y. E. (2019). Clinical and microbiological characteristics of non-tuberculous mycobacteria diseases in Singapore with a focus on pulmonary disease, 2012-2016. BMC Infect. Dis. 19, 436. doi: 10.1186/s12879-019-3909-3, PMID: 31101082 PMC6525426

